# Assessment of Diverse Solid−State Accelerated Autoxidation Methods for Droperidol

**DOI:** 10.3390/pharmaceutics14061114

**Published:** 2022-05-24

**Authors:** Jayant Iyer, Isha Saraf, Andrew Ray, Michael Brunsteiner, Amrit Paudel

**Affiliations:** 1 Research Center Pharmaceutical Engineering GmbH (RCPE), 8010 Graz, Austria; jayant.iyer@rcpe.at (J.I.); isha.saraf@rcpe.at (I.S.); michael.brunsteiner@rcpe.at (M.B.); 2 Global Product Development, Pharmaceutical Technology & Development, AstraZeneca, Macclesfield Campus, Macclesfield SK11 8LF, UK; andrew.ray@astrazeneca.com; 3 Institute of Process and Particle Engineering, Graz University of Technology, 8010 Graz, Austria

**Keywords:** solid−state stress, autoxidation, AIBN, pre−milled PVP K−60, RapidOxy^®^

## Abstract

The present study aimed to investigate methods for accelerating autoxidation of crystalline drugs in the solid-state that can potentially predict real−time stability. Solid droperidol (DPD) was selected as the model drug. A common free−radical initiator, 2,2′−azobisisobutyronitrile (AIBN), was used to induce autoxidation in solutions. AIBN decomposes at elevated temperatures to yield carbon−centred cyano−isopropyl free radicals that can auto−oxidize neighboring drug molecules. Although the reaction of AIBN is relatively straightforward in solution, it is less so in solids. In this study, we used solid AIBN mixed with DPD powder in the presence and absence of pressurized oxygen headspace. Samples were prepared directly in the form of binary mixtures with DPD and additionally in the form of powder compact/pellet with DPD. The main challenge in carrying out the reaction was related to the preservation of AIBN at elevated temperatures due to the disintegration of the pellet containing the latter. A commercially available free−radical coated silica particle (i.e., 2,2,6,6−tetramethyl−1−piperinyloxy (TEMPO) or (SiliaCAT^TM^ TEMPO)) was tested as a potential stressor, but with limited success to induce autoxidation. The most valuable results were obtained when a physical mixture of pre−milled PVP K−60 containing free radicals and DPD was exposed to elevated oxygen−temperature conditions, which yielded significant degradation of DPD. The study highlights the practical challenges for conducting accelerated solid−state stress studies to assess the autoxidation susceptibility of drugs using traditional free−radical initiators and presents a proof of application of milled PVP with free−radical as a potential alternative.

## 1. Introduction

The autoxidation of pharmaceuticals is one of the major degradation routes, often compromising the chemical stability of drug products [1]. Autoxidation in pharmaceuticals typically proceeds in the presence of peroxy radicals generated by the reaction of molecular oxygen with organic free radicals. The organic free−radical is usually a transition metal or an excipient impurity present in trace amounts and acts as an initiator to catalyze the reaction. Once initiated by the free radicals, the reaction proceeds automatically by sequential abstraction of hydrogen atoms from the substrate (drug molecule); hence, they are termed “autocatalytic”. Commonly, autoxidation results in the formation of degradation products (DPs) that are carcinogenic or genotoxic thus, affect the quality and safety of the drug product. Moreover, the reaction is challenging to predict due to the complexity of the reaction pathway and also because it involves molecular oxygen that is ubiquitous in the environment [2,3].

Drug stability is an important safety, efficacy, and quality consideration when developing new drug candidates. Accelerated stability testing of drugs serves as a valuable alternative to long−term stability testing as it shortens the time taken for the submission of registration dossiers. Forced degradation studies are frequently conducted on new drug candidates to quickly evaluate the risk of formation of DPs and impurities generated under long−term or accelerated storage conditions [4]. Previous work from Modhave et al. highlighted the use of PVP−H_2_O_2_ complex in powder state as a solid−state forced−oxidation stressor for vortioxetine hydrobromide [5]. Although several reports have discussed the use of high oxygen/air pressures to induce autoxidation, there is an open question regarding the rate of reaction, selectivity, and the involved mechanism in the case of solid drugs [6,7,8]. Free−radical initiators such as 2,2′−azobisisobutyronitrile (AIBN), 2,2′−azo bis (2−amidinopropane) dihydrochloride (AAPH), 2,2′−azobis(2,4−dimethylvaleronitrile) (AMVN), 4,4′−azobis(4−cyanovaleric acid) (ACVA) are commonly used to induce autoxidation in a solution−state [9,10], but these stressors have been seldom used in a solid−state [11]. AIBN has been widely used as an autoxidation stressor in solution−state and is often a good predictor of long−term degradation, e.g., tetrazepam tablets [12]. Recently, Dana et al. reported a detailed mechanism of the free−radical formation by AIBN in solution [13]. Another report on using AIBN as a stressor in solid−state suggests applying pressurized oxygen conditions at 25 °C under open conditions for a week in selected drug candidates [14]. However, a clear description of experimental methodologies for the use of AIBN in solid−state under high oxygen pressures remains elusive. Apart from azo radical−initiators, a stressor that has been widely used to induce oxidation of alcohols to acids is 2,2,6,6−tetramethyl−1−piperinyloxide (TEMPO) [15,16]. Considering the selectivity of reactions and a plethora of factors such as conformation, crystal lattice disorder, surface area, polymorphism, surface energy, etc., the reactions in solid−state may not resemble the ones occurring in solution−state [17,18]. Moreover, autoxidation in solid−state may involve an uncertain induction time, where there might be minimal to no reaction, thereby complicating the possibility of predicting the reaction pathway [19].

The overall objective of the present study was to induce autoxidation in the solid−state of a model drug, droperidol (DPD), utilizing diverse solid stressors, namely AIBN, SiliaCAT^TM^ TEMPO, and pre−milled poly−vinylpyrrolidone K−60 (PVP K−60) under elevated temperature and pressure. The key emphasis was placed on reducing the time for autoxidation in solid−state from a few weeks to days. These stressors were employed in the form of a physical binary mixture or pellets with DPD. The samples generated during stress studies were analyzed using ultra−high−performance liquid chromatography (UPLC) and mass spectrometry. The study highlights key challenges in conducting the accelerated solid−state autoxidation with AIBN and SiliaCAT^TM^ TEMPO. Alternatively, radical−enriched pre−milled PVP K−60 was found to be a potential stressor in conducting accelerated drug autoxidation in the solid-state within 48 h.

## 2. Materials and Methods

### 2.1. Materials

2,2′−Azobisisobutyronitrile (AIBN) and Droperidol (DPD) were purchased from Merck (Vienna, Austria). 2,2,6,6−tetramethyl−1−piperinyloxy silica gel (SiliaCAT^TM^ TEMPO) was purchased from Silicycle Inc. (Quebec, Canada), while PVP K−60 (with an average molecular weight of 360,000 Da) was procured from Sigma Aldrich (Vienna, Austria). Ultrapure water was obtained from a TKA water purification unit. HPLC grade Methanol was procured from VWR Chemicals (Dresden, Germany). Buffer salts and other chemicals were of analytical reagent grade.

### 2.2. Methods

#### 2.2.1. General Workflow for Testing Autoxidation

**Figure 1:**
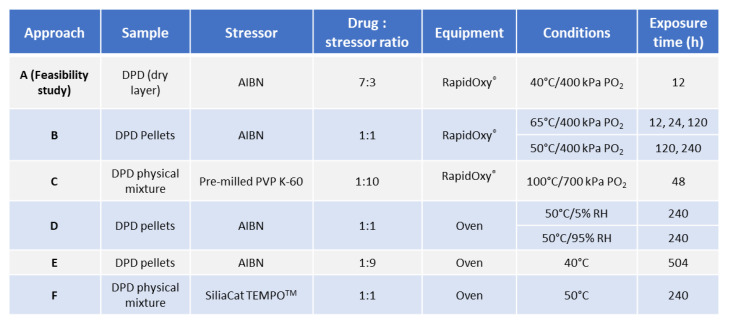
General workflow to test autoxidation in solid drugs.

Figure 1 depicts the general workflow that was used to induce autoxidation in solid DPD. The aim was to generate autoxidation in DPD.

#### 2.2.2. Investigation of Solid−state Degradation in DPD Using RapidOxy^®^

An oxidative stress study on solid DPD was performed using a RapidOxy^®^ 100 device from Anton Paar (Anton Paar ProveTec GmbH, Ostfildern, Germany) with an instrument number 1.03.1378.136 and serial number 60062611. The operating system was version 1.249. Various combinations of temperature and exposure time were attempted to induce degradation. These approaches are described in the following sections.

**Approach A** (feasibility study): The small−scale experiment was conducted in DSC pans. Solid AIBN was dissolved in acetone to yield a 0.3 mg/mL solution and poured into DSC pans to form a dry layer after evaporation (replicates N = 3). Over the dry layer of AIBN, DPD was topped over to give AIBN:DPD in a 3:7 ratio finally. Replicate samples were placed into DSC pans and loaded into RapidOxy^®^ at 40 °C/12 h/400 kPa.

**Approach B**: Solid DPD and AIBN in a 1:1 ratio were physically blended and compressed into pellets (replicates, N = 3). Compaction was carried out using a hydraulic press at 4 tonnes of pressure and a dwell time of 60 s. Such pellets were then loaded in RapidOxy^®^ at 65 °C and 400 kPa at three exposure times of 12, 24, and 120 h, respectively. Replicate pellets were also loaded at 50 °C and 400 kPa at 120 and 240 h, respectively.

**Approach C**: A physical mixture of pre−milled PVP K−60 and DPD (10:1) was loaded into RapidOxy^®^ (100 °C/48 h/700 kPa). PVP K−60 was chosen as the stressor due to the presence of higher free radicals. Three different povidone grades (PVP K−30, PVP K−60, and PVP K−90) were screened for free−radical content before and after ball−milling using electron paramagnetic resonance (EPR) spectroscopy. Ball milling of povidones was carried out using Mixer Mill MM400 (Retsch, Germany). Approximately 3000 mg of powder was placed in a 50 mL milling jar. A single stainless−steel ball of 2.0 cm diameter was used, and milling was carried out at 25 Hz for 60 min. The EPR measurements were performed on Magnettech Miniscope MS300 benchtop X−band EPR spectrometer (Bruker, Frankfurt, Germany). Approximately 25 mg sample was placed in a glass sampling tube to record the EPR spectra. The center field and sweep width were kept at 3396.61 G and 100 G, respectively, while the attenuation was 10 dB. Ten scans were acquired within a period of 60 s. Data analysis was conducted using Multiplot software (version 1.5, Champaign, IL, USA) and Microsoft Excel (version 2108, Washington, DC, USA).

A kinetic study was carried out by using a physical mixture of DPD and pre−milled PVP K−60 (1:10) at different time points such as 6, 9, 24, and 48 h using the same conditions (100 °C/700 kPa) in RapidOxy^®^ (replicates N = 2). Statistical analysis, including the t−test, was performed on the degradation values using Excel software (Microsoft Office, version 2108, Washington, DC, USA). The first array was kept as the exposure time in RapidOxy^®^ and the second array was the extent of degradation, and a two−tailed distribution of type two−sample equal variance was used to determine the *p*−values.

Four different sets of control experiments were conducted to evaluate the role of the stressor (pre−milled PVP K−60), nitrogen gas pressures, and ambient environment pressures. The temperature and exposure times were kept at 100 °C and 48 h in all the cases and three replicates were used in each experiment. In the first set, control samples of DPD crystals (devoid of pre−milled PVP K−60) were exposed to 100 °C/700 kPa for 48 h in RapidOxy^®^ to evaluate drug degradation under the selected temperature and pressure. In the second set, a control experiment was conducted by the use of nitrogen gas pressure instead of oxygen gas pressure in RapidOxy^®^. The rest of the exposure conditions were kept the same. The third and fourth sets of experiments were conducted under ambient atmospheric pressures. In the third set, pellets were prepared using a hydraulic press comprising the physical mixture DPD:pre−milled PVP K−60 (1:10). These pellets were placed in glass vials and exposed to 100 °C in an oven (Binder, Germany). In parallel, DPD crystals were placed in a glass vial as the fourth control set.

To verify the formation of DPs by autoxidation, a solution−state stress study was carried out on DPD by using 10 mM AIBN as the stressor and exposing it at 50 °C for 3 h, while 20 mM AIBN stock solution was prepared in ACN. 1000 µg mL^−1^ drug stock solution was prepared in 7:3 (MeOH:water). Finally, both stock solutions were combined in equal proportions so that the final concentration of the drug was 500 µg mL^−1^. Solutions devoid of AIBN and the drug were kept in parallel as controls.

#### 2.2.3. Investigation of Solid−state Degradation in DPD Using an Oven

The feasibility of checking oxidation in solid DPD in the absence of an oxygen pressurized system was conducted in the oven.

**Approach D**: To assess the feasibility of degradation without the elevated oxygen pressure conditions, replicate pellets containing a ratio of AIBN:DPD 1:1 were placed in an oven (under atmospheric oxygen pressure) at 50 °C/5% RH for 240 h, respectively. Moreover, the impact of higher humidity (i.e., at 50 °C/95% RH for 240 h) on oxidation was also studied by placing replicate pellets in a desiccator filled with ultra−pure water, which was then loaded into an oven. Individual pellets composed of DPD and AIBN alone were also placed in parallel as controls.

**Approach E**: aimed to use a higher composition of AIBN than DPD, i.e., in a 1:9 ratio of DPD:AIBN to form pellets as previously described. Such pellets were placed in the oven at 40 °C for 504 h. A lower temperature was chosen because the pellets’ higher AIBN proportion could accelerate the physical disintegration.

**Approach F**: Rather than using AIBN, which needs pre−activation to form radicals and induce degradation, the aim was to use a commercial free−radical stressor that does not need pre-activation. For this purpose, SiliaCAT^TM^ TEMPO was used. Due to the fine particle nature of SiliaCAT^TM^ TEMPO, approaches to form compacts/pellets failed. Hence, the reaction could only be made with a binary mixture of TEMPO and DPD in a 1:1 ratio, and the sample was charged in the oven at 50 °C for 240 h.

#### 2.2.4. Liquid Chromatography

LC analyses were performed using Waters (Milford, CT, USA) Acquity^TM^ UPLC H−Class system equipped with a photodiode array (PDA) detector and a quaternary pump. Empower 3.0 software was used for data acquisition and data processing. The reversed−phase liquid chromatographic method was developed using a C18 UPLC BEH column (column id: 03423914225174), 1.7 µm, 2.1 mm (*i.d.*) × 100 mm (length). The aqueous mobile phase was A = 10 mM Ammonium acetate (pH 5.05) adjusted using acetic acid, and the organic mobile phase was B = MeOH. Flow rate was kept constant at 0.35 mL/min with gradient programme T_min_/A%; T_0.0_/70; T_2.0_/70; T_10.0_/15; T_12.01_/70 with 2 min end equilibration. The column temperature was set at 40 °C, while the autosampler temperature was maintained at 25 °C during analysis. The injection volume was 1 µL, and the detection wavelength was kept at 235 nm. The method was specific for determining the main component in the presence of DPs. The sample was prepared in diluent comprising MeOH:water (7:3 *v/v*) to yield a final concentration of 500 µg mL^−1^. Another UPLC method was used for samples containing polymer (pre−milled PVP K−60), which consisted of C8 UPLC BEH column (column id: 03633020315173), 1.7 µm, 2.1 mm (i.d.) × 100 mm (length). All the other chromatographic conditions were kept the same. The area under the curve of peaks (AUC) was used to report the extent of degradation of DPD in comparison to peak areas of DPs. Peaks below a detection threshold (area) of 0.05% were not integrated and thus are not reported. Before injection, samples containing PVP K−60 were filtered with a 0.2 µm Nylon membrane filter.

#### 2.2.5. Liquid Chromatography−Single Quadrupole Mass Spectrometry (LC−SqD MS)

LC−SqD MS of a representative sample after RapidOxy^®^ treatment was performed to identify the formed DPs. All samples were measured with the Waters Acquity^TM^ UPLC−SqD MS instrument (Milford, MS, USA) using Waters Empower 3.0 software for data acquisition and processing. The LC methods described in the previous section were the same. A full scan of masses in the m/z range of 150 to 800 Da was performed with a cone voltage of 30 V and capillary voltage of 3 kV. The desolvation temperature and ion source temperature were 400 °C and 150 °C, while the desolvation gas flow (nitrogen) was kept at 600 L/h, respectively. Data were acquired in electrospray ionization (ESI) positive and negative modes. The detailed structure elucidation of DPs was not the main objective of the present study, and the line spectra corresponding to the DPD and DPs were compared to the literature.

## 3. Results

### 3.1. Approach A−Feasibility Study

The physical and visual assessment of DPD samples topped over the AIBN layer and when exposed to RapidOxy^®^ in DSC pans, did not reveal any change in physical appearance. In addition, there was no degradation when analyzed using UPLC, probably due to the lack of inter−particle contact on the surface and limited availability of volatile radicals at elevated temperatures of 50 °C.

### 3.2. Approaches−B, D, E, and F

#### 3.2.1. Assessment of Pellets Exposed in RapidOxy^®^

**Figure 2:**
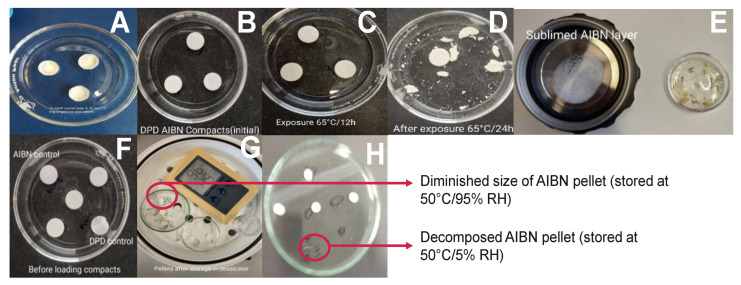
Images showing autoxidation approaches with DPD:AIBN (1:1) pellets exposed to different conditions. (**A**) Feasibility approach with AIBN layer topped with DPD in DSC pans before exposure in RapidOxy^®^; (**B**) original appearance of DPD:AIBN pellet before exposure in RapidOxy^®^; (**C**) appearance of pellets after exposure in RapidOxy^®^ (65 °C/12 h/400 kPa); (**D**) disintegrated pellets after exposure in RapidOxy^®^ (65 °C/24 h/400 kPa); (**E**) AIBN layer deposited on the lid (left) and disintegrated pellets (right) after exposure in RapidOxy^®^ (65 °C/120 h/400 kPa); (**F**) original appearance of pellets before exposure in the oven; (**G**) appearance of pellets after exposure in the oven (50 °C/95% RH/240 h); (**H**) Appearance of pellets after exposure in the oven (50 °C/5% RH/240 h).

Figure 2B–E shows the physical appearance of compressed pellets before and after RapidOxy^®^ exposure. As shown in Figure 2C, exposures at 65 °C for 12 h did not make any significant difference to the original appearance of pellets, but extending exposure time to 24 h disintegrated the pellets (Figure 2D). The LC analysis of samples exposed to RapidOxy^®^ at 65 °C for 12 h and 24 h yielded the same area percent (99.51%), indicating no progress in the overall degradation. Extending the exposure time to a period of 120 h at 65 °C led to the disintegration of pellets accompanied by the appearance of yellow color residue. This event indicated a physical and chemical change (Figure 2E). An image in Figure 2E also displays the sublimed AIBN (from the disintegrated pellets) deposited on the lid of the RapidOxy^®^ device.

LC analysis of this sample yielded a higher degradation (1.71% peak area reduction than pure API) as compared to previous experiments. However, considering the method’s low feasibility due to pellets’ disintegration, this approach was discontinued.

#### 3.2.2. Assessment of Pellets Exposed to Oven

**Figure 3:**
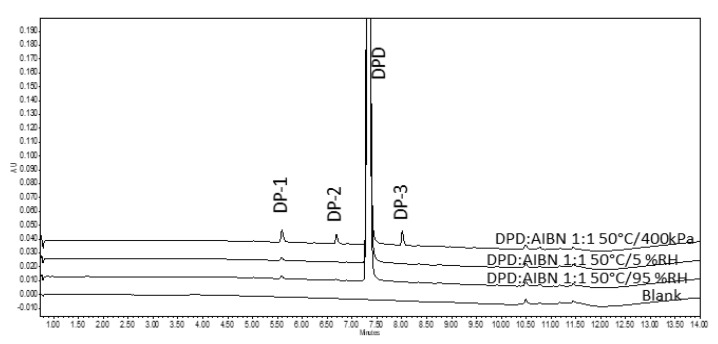
Chromatogram showing the formation of DPs in DPD:AIBN compacts when exposed to different oxidative conditions and an enrichment of DPs when exposed under high oxygen pressures (400 kPa) in RapidOxy^®^.

**Figure 4:**
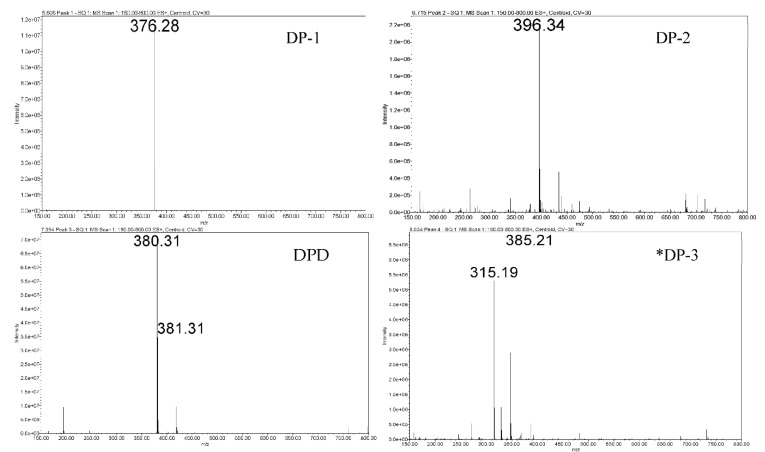
LC−SqD MS characterization of DPD and its DPs. (* The structure of DP-3 could not be justified based on the available data).

Sample pellets containing DPD:AIBN in 1:1 ratio were exposed at 50 °C/5% RH and 50 °C/95% RH in a desiccator for 240 h and appeared pale yellow. It was also noted that the AIBN pellet (control) had disappeared entirely from the petri dish at the end of 240 h exposure to 50 °C/5% RH, while it got diminished in size after exposure at 50 °C/95% RH (shown in Figure 2G,H). Upon analysis of the sample pellets, exposed in the oven for 240 h (including the ones in the desiccator) (described in Section 3.2.1) by the LC method, it was found that there was less degradation as compared to the disintegrated pellets in RapidOxy^®^ exposed for 240 h. However, the same DPs were formed in all the samples (see Figure 3). LC−SqD MS characterization of the samples (Figure 4) resulted in the identification of two DPs of DPD, designated DP−1 (0.76 RRT) and DP−2 (0.91 RRT). The molecular ion of DPD is m/z 380.31, corresponding to a molecular formula C_22_H_22_N_3_O_2_F, while that for DP−1 and DP−2 are 376.28 and 396.34, respectively. It is inferred here that DP−1 could result from dehydrogenation (loss of four hydrogens) of the DPD, resulting in the formation of a structure with an aromatized pyridine ring (DPD Impurity−C European Pharmacopoeia (EP)) corresponding to molecular formula C_22_H_18_N_3_O_2_F. DP−2 includes the addition of 16 m/z to the molecular ion of DPD, indicating the formation of an N−oxide at the tertiary nitrogen atom in the ring (DPD Impurity−D EP) corresponding to a molecular formula of C_22_H_22_N_3_O_3_F [20]. Apart from the two DPs, an additional peak eluted after the DPD peak in LC (1.09 RRT). However, the structure of this peak could not be established based on the m/z of the DPD.

The sample pellets, which comprised DPD:AIBN in a 1:9 ratio (**Approach E**) also underwent disintegration at 40 °C (albeit slowly) indicating that the use of higher AIBN content with simultaneous reduction of temperature would not be feasible to accelerate the degradation.

#### 3.2.3. Approach C (Binary Mixture of Pre−milled PVP K−60 and DPD (10:1))

**Figure 5:**
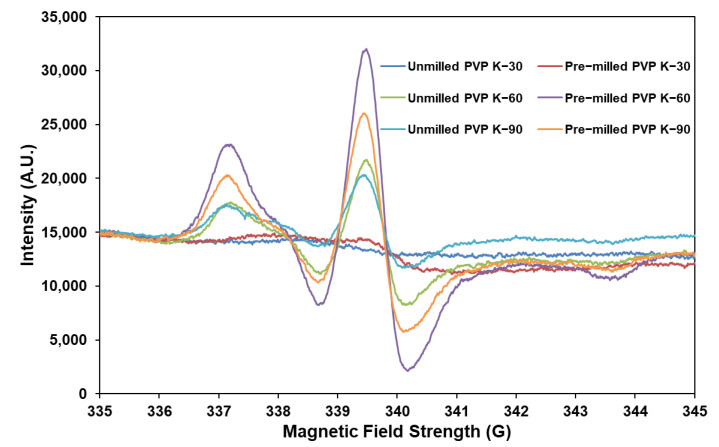
Enhancement of hyperfine signals in pre−milled povidones.

Using povidone excipients as the stressor was based on the observation of free−radical signals in the EPR spectrum and considering that autoxidation of drugs is catalyzed in the presence of free radicals. It was experienced that milling povidones (grades K−30, K−60, and K−90) increased the formation of radicals as indicated by an increased hyperfine EPR signal intensity (Figure 5). This increase was the most prominent in PVP K−60 grade as compared to PVP K−30 and PVP K−90. Hence, pre−milled PVP K−60 was used as the stressor in 10−fold weight to DPD powder.

**Figure 6:**
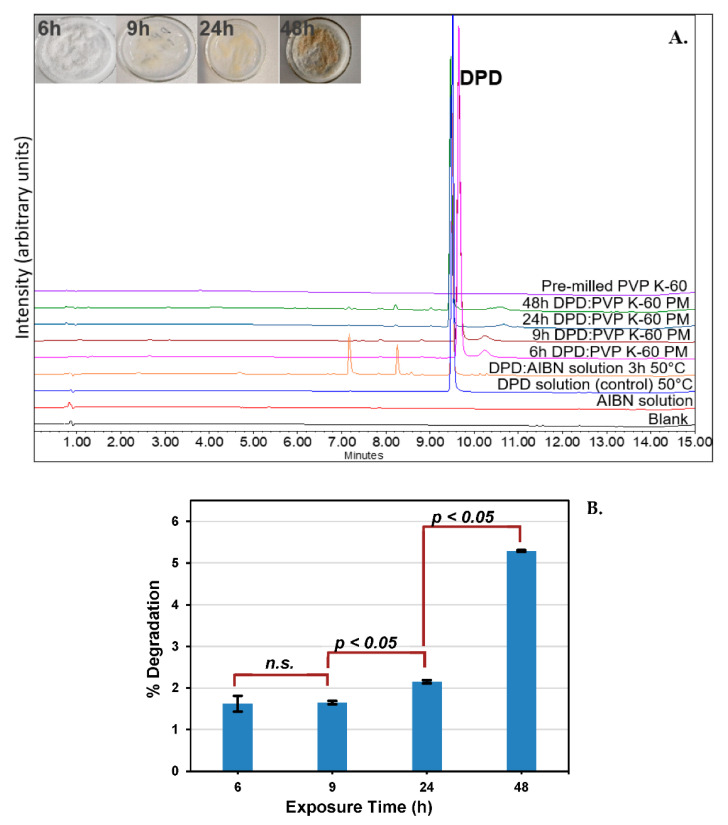
(**A**). Overlaid chromatogram of DPD samples after exposure in RapidOxy^®^ indicating enrichment of DPs. (PM denotes the physical mixture of DPD and pre−milled PVP K−60 in a 1:10 ratio) (pictures in the inset show the sample appearance at different exposure durations); also shown in the overlay is a comparison with a solution−state stress study carried out with AIBN, indicating the formation of similar products; (**B**). Bar chart showing the test of significance in the observed degradation values of PMs as a function of exposure time in RapidOxy^®^; n.s. indicates the *p*−value was not significant.

DPD was found to degrade up to 5.29 ± 0.02% (94.70% AUC) when exposed to pre−milled PVP K−60 for 48 h/100 °C/700 kPa in RapidOxy^®^. A replication of the experiment gave 94.73% AUC, which indicated that the method was reproducible (data not shown). Experiments at lower time points were also conducted to verify the formation of DPs and assess the kinetics (Figure 6A). Statistical analysis (*t*−test) was conducted to check the significance of measured degradation values (Figure 6B). The calculated *p*−value from 6−9 h exposure was 0.06, whereas between 9−12 h and 12−24 h, the values were 0.031 and 0.047, respectively, indicating a statistically significant result (*p* < 0.05).

**Figure 7:**
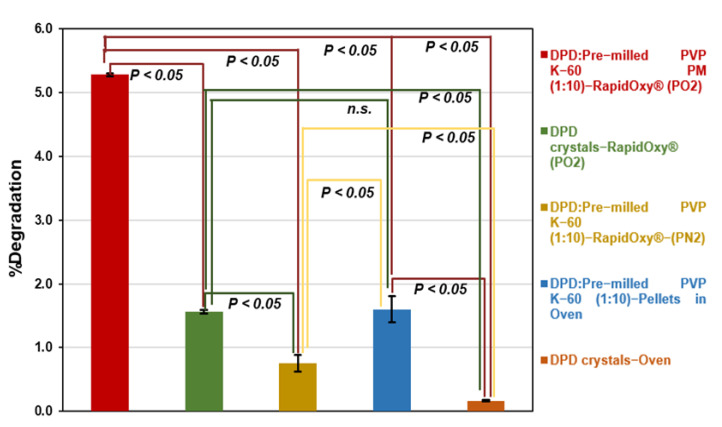
Evaluation of differences in oxidative degradation of DPD samples at 100 °C and 48 h conditions (green, yellow, blue, and orange bars indicate the degradation in control samples). Test of significance among degradation values is indicated by *p*−value (*p* < 0.05 indicates the difference in degradation was statistically significant) whereas, n.s. indicates the *p*−value was not significant.

Analysis of control samples did not reveal remarkable degradation as compared to the PM exposed to RapidOxy^®^. As shown in Figure 7, DPD crystals (alone) exposed under the same conditions in RapidOxy^®^ showed almost three−fold lesser degradation (1.56 ± 0.03%) than the PM, indicating the role of a stressor (pre−milled PVP K−60) in inducing oxidation. Repetition of the experiment containing DPD:pre−milled PVP K−60 (1:10) PM under nitrogen pressures instead of oxygen pressures also resulted in a much lesser degradation of 0.75 ± 0.13%, highlighting the presence of oxygen in inducing autoxidation. It also implies that high pressure alone does not lead to degradation.

Similarly, the exposure of pellets containing the PM as mentioned above under atmospheric pressure (in an oven) resulted in a degradation of 1.60 ± 0.21% (98.40% AUC), indicating that degradation can proceed in the presence of stressor (pre−milled PVP K−60) albeit to a lower extent as compared to pressurized oxidation setup. Negligible degradation of 0.16 ± 0.01% was observed when the DPD crystals (alone) were exposed in an oven at 100 °C under atmospheric pressure. Statistical analysis of degradation values indicated that the difference was statistically significant in all the control samples (*p*−values were below 0.05). An exception to this was between the DPD crystals exposed in RapidOxy^®^ under accelerated pressure and the DPD:pre−milled PVP K−60 (1:10) pellets exposed in the oven (Figure 7).

## 4. Discussion

An accelerated experimental method to access solid−state degradation of drug substances and drug products is important yet not straightforward. There have been few efforts to develop solid−state stress methods, with the most elaborate studies done for drug hydrolysis in solid−state [21]. Such studies for autoxidation assessment are rarely reported in solid−state, albeit many studies and methods exist for solution−state studies. Our above−discussed studies indicate the practical difficulties encountered while using AIBN (a standard solution−state stressor) as the solid−state stressor for drug autoxidation. Following an initial feasibility study (approach A), where there was no degradation, our approach shifted to form pellets. It is believed that compaction would enhance the contact surface among particles of AIBN and drug, thereby leading to reaction. A principal challenge in pellets was to retain the AIBN at elevated experimental temperatures. An increase in temperatures to 65 °C leads to the disintegration of pellets due to a well−known thermal activation of AIBN. Another approach E, was aimed to accelerate the degradation by using AIBN in higher composition, i.e., DPD:AIBN in a 1:9 ratio at 40 °C and exposed for 504 h. However, it was observed that the pellets disintegrated even at 40 °C (albeit slowly).

The thermal decomposition characteristics of AIBN in solid−state are well documented by Lazar et al. [22]. The authors propose a two−step decomposition mechanism of AIBN due to the nucleation of DPs (ketenamine and tetramethyl succinonitrile) in the solid crystal phase. They speculate that ketenamine may act as a decomposition initiator. Another work from Li et al. discusses the quasi−autocatalytic decomposition mechanisms of AIBN below its melting point [23]. The authors speculate that a sublimation or physical phase transition step overlaps with its chemical reaction step, where the endothermic and exothermic events compete. They reported a DSC scan (10 K min^−1^) of solid AIBN, which revealed an endothermic peak around 70–80 °C, indicating a phase transition in the crystal. This event was followed by an endotherm highlighting the melting of AIBN crystals. Soon after the melting event, a decomposition step (in melt state) followed. At significantly lower heating rates (0.01 K min^−1^), the decomposition (exotherm) step begins at lower temperatures and hides the melting event.

It is assumed that the AIBN is continually dissociated at temperatures above 40 °C to yield volatile free radicals that leave the pellet matrix before the reaction occurs. Methods of confining AIBN inside the pellet would necessitate one to use ambient room temperatures (25−30 °C), leading to exceedingly prolonged exposure times to induce degradation.

LC−SqD MS analysis of the 240 h exposed pellet samples in RapidOxy^®^ and oven at the same temperature of 50 °C, still at different oxygen partial pressures, indicated that the same DPs were formed in the oven (ambient atmospheric pressure) as were formed in RapidOxy^®^ (elevated oxygen pressure). Although a greater extent of degradation was observed for the sample in RapidOxy^®^ (240 h exposure), the pellets disintegrated. The cumulative percentage degradation achieved in all the approaches mentioned above is shown in Table 1.

To circumvent the issues with the activation required for AIBN to form free radicals, the approach shifted to use a free−radical, readily available in solid−state and marketed commercially as SiliaCAT^TM^ TEMPO. However, a binary mixture of DPD and SiliaCAT^TM^ TEMPO did not yield degradation. Moreover, the extremely fine particle size of SiliaCAT^TM^ TEMPO did not allow compaction with DPD.

More useful outcomes were observed in approach C, comprising a binary mixture of pre−milled PVP K−60 in a ratio of 10 folds weight to that of DPD yielded a significant degradation in RapidOxy^®^. The ratio was obtained based on preliminary experiments. Kinetic analysis at lower exposure time points indicated that the degradation rate was not linear, as was expected (for typical solid−state reaction). The benefits of using pre−milled PVP K−60 over AIBN in catalyzing autoxidation are manifold. In addition to enriching DPs within a shorter time, the reaction was non−invasive (i.e., without any thermal decomposition of stressor).

Moreover, the use of a pressurized oxidation device (RapidOxy^®^) enabled exposures at higher temperatures (100 °C) with elevated oxygen pressure (700 kPa). This approach entailed that the sample could be analyzed in the same physical state as it was during the initial time before exposure. It is expected that the degradation is more selective to oxidation than the reactions carried out under ambient atmospheric conditions. Statistical evaluation of the degradation values indicated that after 9 h exposure, the results were significant (*p*−value < 0.05) (Figure 6). However, below 9 h the difference in degradation values was not statistically significant. This result indicates the possibility of induction time before the reaction accelerates to yield a significant degradation and is expected for autoxidation occurring in the solid−state [24,25].

A comparison of formed DPs with the solution stress study carried out using an azo free−radical initiator AIBN indicates the formation of similar DPs in the solid−state (see Figure 6). Although not every DP matches the ones found in solution−stress 4 DPs, are formed in both solid and solution−states. This outcome is expected in the solid−state, as there might be the role of solid−state properties governing nucleation and growth of DPs on crystal face apart from the differences in conformations as compared to a solution phase. The control solution of the drug without AIBN exposed at the same temperature (50 °C) does not indicate any DPs. Hence, it concludes that the drug is practically thermo−stable under the tested conditions.

Control experiments (Figure 7) in solid−state included the exposure of the drug alone under the described conditions in pressurized oxygen condition (RapidOxy^®^) and at ambient oxygen pressure (in an oven). There was comparatively a higher degradation in RapidOxy^®^ in this case; however, it was much lesser than the PM. This result indicates the catalytic role of radical−containing stressors in leading to autoxidation. When the reaction involving PM was performed by using a nitrogen gas pressure instead of oxygen gas pressure, there was much lesser degradation indicating the reaction’s selectivity to oxygen. Additionally, the use of pellets containing the PM exposed to ambient atmospheric conditions also leads to degradation, albeit to a much lesser extent than the RapidOxy^®^. All the described results point to the presence of oxygen and the role of higher oxygen pressures combined with free−radical enriched stressor to accelerate autoxidation in the solid-state. Figure 7 also indicates that the degradation values were statistically significant in all the cases except between the control samples; DPD crystals (degradation in RapidOxy^®^) and the DPD:pre−milled PVP K−60 (1:10) pellets (degradation in the oven).

The role of elevated oxygen pressures in inducing autoxidation is already reported in the literature [6,7]. A thorough elucidation of mechanism(s) and/or the rate of autoxidation of solid drugs in pressurized devices remains to be done. Although further investigation of the results and the oxidation mechanism is in progress, the initial results presented herein are promising. Future work in this research is directed to the study of autoxidation of compounds representing a range of C−H bond dissociation energies (BDE). A report details three methods to measure C−H BDEs however, none of them appear to be experimentally feasible in a general laboratory setting [26]. Due to these reasons, in multiple reports, C−H BDE has been determined by in−silico calculations to predict the autoxidation of drugs [3,8,27,28,29]. However, the report entailing the direct applicability of C−H BDE to experimental degradation of drugs is rare. Therefore, this would be a follow−up study.

## 5. Conclusions

The paper describes a fast and novel method for conducting autoxidation of solid drugs based on a pressurized oxidation device in a physical combination with pre−milled PVP K−60. We describe practical challenges when using two different solid stressors, AIBN, and SiliaCAT^TM^ TEMPO, in conducting autoxidation of solid drugs making their choice unfeasible. However, the use of a binary mixture comprising pre−milled PVP K−60:DPD (10:1) in a pressurized oxidation device enables the enrichment of DPs quickly under accelerated temperature exposure. Analysis of the exposed DPD sample with LC−MS revealed the formation of autoxidation products. Overall, this study provides a practical benefit of the developed method for the early−stage assessment of novel candidates’ autoxidative (in)stability. However, the applicability of this approach needs to be evaluated, ensuring the conditions such as concentration of PVP, temperature, and duration, so that the autoxidation is representative of that generated under the International Council for Harmonization of Technical Requirements for Pharmaceuticals for Human Use (ICH) conditions for a range of drug molecules.

## Figures and Tables

**Table 1:** Experimental results indicating degradation with approaches containing AIBN:DPD (in replicates N = 3).

Approach	Method	AIBN:DPD Ratio	Conditions	%Degradation (100-%Area of DPs)
A	RapidOxy^®^	1:1	40 °C/12 h/400 kPa	NIL
B *	RapidOxy^®^	1:1	65 °C/12 h/400 kPa	0.39 ± 0.14
			65 °C/24 h/400 kPa	0.42 ± 0.10
			65 °C/120 h/400 kPa	1.57
			50 °C/120 h/400 kPa	0.67
			50 °C/240 h/400 kPa	1.71
D	Oven	1:1	50 °C/5% RH/240 h	0.34 ± 0.11
			50 °C/95% RH/240 h	0.43 ± 0.08
E	Oven	9:1	40 °C/5% RH/504 h	0.51 ± 0.01

* Disintegration of pellets in Approach B did not permit analysis of intact replicates.

## Data Availability

The supporting data (including raw data) shall be archived and provided upon request.
